# ROS in the Male–Female Interactions During Pollination: Function and Regulation

**DOI:** 10.3389/fpls.2020.00177

**Published:** 2020-02-28

**Authors:** Ming Jun Zhang, Xian Sheng Zhang, Xin-Qi Gao

**Affiliations:** National Key Laboratory of Crop Biology, College of Life Sciences, Shandong Agricultural University, Taian, China

**Keywords:** reactive oxygen species, stigma, style, female gametophyte, pollen

## Abstract

The male–female interactions in pollination mediate pollen hydration and germination, pollen tube growth and fertilization. Reactive oxygen species (ROS) derived from both male and female tissues play regulatory roles for the communication between the pollen/pollen tube and female tissues at various stages, such as pollen hydration and germination on the stigma, pollen tube growth in the pistil and pollen tube reception in the female gametophyte. In this minireview, we primarily summarize the recent progress on the roles of ROS signaling in male–female interactions during pollination and discuss several ROS-regulated downstream signaling pathways for these interactions. Furthermore, several ROS-involved downstream pathways are outlined, such as Ca^2+^ signaling, cell wall cytomechanics, the redox modification of CRP, and cell PCD. At the end, we address the roles of ROS in pollen tube guidance and fertilization as future questions that merit study.

## Highlights

ROS as signal function in male–female interactions during pollination, including pollen hydration and germination on the stigma, pollen tube growth in the pistil and pollen tube reception in the female gametophyte.

## Introduction

Pollination is a critical step for sexual plant reproduction. After landing on the stigma, the pollen undergoes adhesion and hydration before it germinates to create a pollen tube. The polar tip growth of the pollen tube guides it through the maternal tissues toward the female gametophyte. On arrival at the female gametophyte, the rupture of the pollen tube releases two sperms in a degenerated synergid cell for fertilization. The interactions between the pollen (pollen tube) and maternal tissues (stigma, style, ovule and female gametophyte) are critical for pollen hydration and germination, pollen tube growth in the pistil tissues, guidance to the female gametophyte, reception of the female gametophyte and sperm-egg cell fusion (Johnson et al., 2019; Lopes et al., 2019). Reactive oxygen species (ROS; e.g., O^2^•−, H_2_O_2_, OH•, ^1^O_2_) in cells that serve as signaling molecules are involved in various biological processes (Waszczak et al., 2018). ROS play roles in plant development, stress responses, and sexual plant reproduction, such as pollen development, pollen tube tip growth, embryo sac development and fertilization (Cárdenas et al., 2006; Liu et al., 2009; Martin et al., 2013; Lassig et al., 2014; Xie et al., 2014; Huang et al., 2019; Jiménez-Quesada et al., 2019). In contrast, under heat stress conditions, the increased ROS in pollen tubes inhibits tube growth, and flavonols control the pollen tube growth and integrity by regulating ROS homeostasis (Muhlemann et al., 2018). During pollination, ROS derived from both the pollen and female tissues are involved in their communications at various stages. In this review, we summarize the recent progress of the ROS signaling roles concentrating on the male–female interactions in pollination.

## ROS Involved In Pollen Hydration and Germination on the Stigma

**Figure 1:**
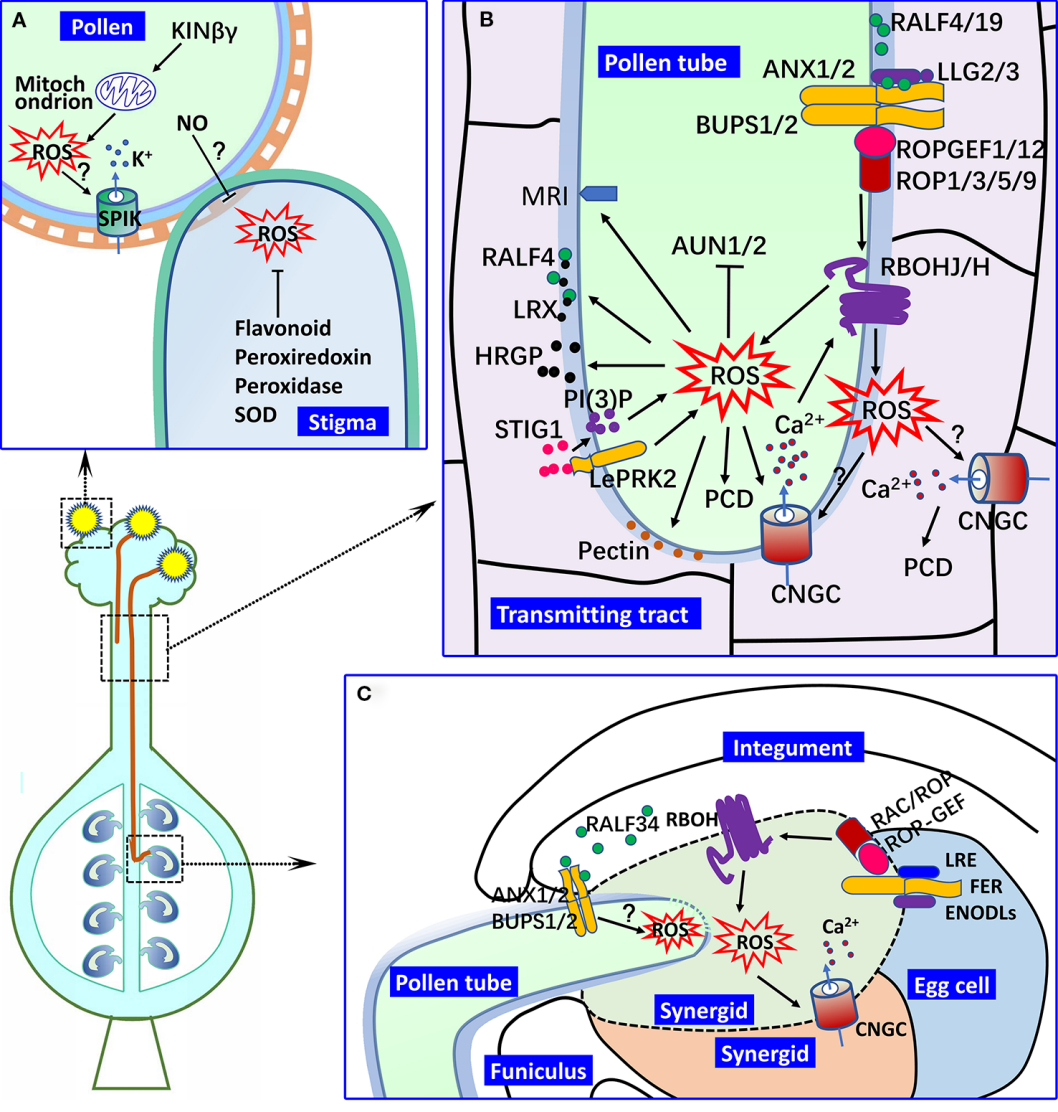
ROS in male–female interactions. **(A)** Pollen hydration and germination on the stigma. In *Arabidopsis* pollen, KINβγ mediates the biogenesis of mitochondrion and ROS levels, which regulate pollen hydration and germination on the stigma. In this process, the expression of *SPIK* might be regulated by ROS signaling, by which ROS signaling mediates K^+^ transport and pollen hydration on the stigma. ROS accumulation is found in receptive stigma, which is important for pollen attachment, but its decrease is required for the germination of compatible pollen. The ROS levels of the stigma are controlled by various oxidoreductase and flavonoids and could also be regulated by NO from the pollen. **(B)** Pollen tube growth in the pistil. NAD(P)H oxidase RBOHH/J-mediated apoplastic ROS production in the growing tip of the pollen tube is important for pollen tube integrity and growth in the pistil by regulating the activities of calcium channels (such as the CNGCs), the secretion of HRGPs (such as LRXs), and the metabolism of wall materials (such as pectin and callose). A type one protein phosphatase (AUN1/2) and plasma membrane-localized receptor-like cytoplasmic kinase MRI also function downstream of ROS signaling in pollen tube growth. The RALF-LLG-BUPS-ANX receptor–ligand interaction is involved in the active regulation of RBOHs and ROS generation, which is mediated by ROPGEFs and ROPs. Pistil-derived STIG1 induces the ROS production of the growing pollen tube in the transmitting tract mediated by LePRK2 and PI(3)P. Apoplastic ROS of the pollen tube might induce PCD of the transmitting tract by mediating CNGC activity and Ca^2+^ signaling. **(C)** Pollen tube reception in the female gametophyte. Pollen tube rupture in the synergid is controlled by ROS from RBOHs in the female gametophyte. LRE and ENODLs serve as the co-receptors of FER to regulate the activity of RBOHs and ROS generation in the synergid, and RAC/ROP might mediate this process. RALF34 primarily derived from the inner integument controls the pollen tube rupture in degenerated synergid by binding to the BUPS/ANX receptor complex in the pollen tube, during which ROS might act downstream of the BUPS/ANX receptor complex. (? indicates the putative regulation.).

Pollen grains undergo adhesion and hydration after landing on the surface of the stigma and germinate to create pollen tubes. The pollen–stigma interaction is critical for pollen adhesion, hydration and germination, and many factors, such as proteins and lipids located on the surface of pollen, have been shown to be involved in this process (Hiscock and Allen, 2008; Dresselhaus and Franklin-Tong, 2013; Doucet et al., 2016). *Arabidopsis* KINβγ is a plant-specific subunit of the SNF1-related protein kinase 1 complex, which functions in the biogenesis of mitochondria and peroxisomes in pollen (Gao X.-Q. et al., 2016). In the null mutant of *Arabidopsis KINβγ*, the ROS levels of the pollen grains are reduced, and the pollen adhesion and hydration on the stigma surface is compromised. Additionally, the ROS signal might regulate the expression of the inward shaker K^+^ channel *SPIK* in pollen, which is important for pollen hydration and germination on the stigma and pollen tube growth in the pistil (Mouline et al., 2002; Li et al., 2017). Thus, the ROS signaling that originates from the interior of the pollen grains mediates the pollen–stigma interactions (**Figure 1A**).

The compromised adhesion and germination of the pollen grains on the non-stigma surfaces indicate that the stigma factors are important for the pollen–stigma interaction (Ma et al., 2012). ROS accumulation is found in the stigmas of various angiosperm species, including *Magnolia* (a primitive angiosperm) and *Arabidopsis* (McInnis et al., 2006; Zafra et al., 2016). Stigma receptivity is correlated with the activity of ROS-related enzymes, such as superoxide dismutase and peroxidase (McInnis et al., 2005; Sharma and Bhatla, 2013), indicating that ROS accumulation in the stigma is a self-regulated process. Recently, the ROS accumulation controlled by flavonoids and the ROS metabolic enzymes were identified in the stigma of ornamental kale (*Brassica oleracea* var. *acephala*), a self-incompatible (SI) species (Lan et al., 2017). The decreased ROS levels in the ornamental kale stigma after treatment with exogenous flavonoid (kaempferol) do not compromise the SI response of the stigma, but the attachment and germination of the compatible pollen is drastically reduced. In contrast, the adherence of pollen grains that trigger the decrease of ROS in the stigma and nitric oxide (NO) from the adhesive pollen as the inducing factor for ROS decrease have been suggested (Hiscock et al., 2007; Serrano et al., 2011; Sharma and Bhatla, 2013), which further supports the suggestion that regulation of the ROS in the stigma is involved in the signaling for pollen–stigma interactions (Hiscock and Allen, 2008; Zafra et al., 2016). Therefore, a possible scenario is that the levels of higher ROS in the mature stigma are favorable for the early stage of the pollen–stigma interaction, e.g., pollen adhesion and hydration, and the decrease in the ROS in the stigma after pollen landing might provide a surrounding for compatible pollen tube growth in the stigma tissue (**Figure 1A**).

## ROS Regulate Pollen Tube Growth in the Pistil

The facilitation of the pollen tube growth in the pistil tissues by the apoplastic ROS has been well studied. *Arabidopsis* respiratory burst oxidase homologs (RBOHs) are plasma membrane-localized NAD(P)H oxidases, which are essential for pollen tube penetration into the transmitting tract of pistil by mediating apoplastic ROS production in the growing tip of the pollen tube (Kaya et al., 2014; Kaya et al., 2015). The pollen tube of the *rbohh,j* double mutant exhibits bursting *in vitro* and retarded growth in the pistil. In contrast, in the self-incompatible pollen tube, the increase in ROS levels triggers programmed cell death (PCD) and the self-incompatibility response (Serrano et al., 2015) (**Figure 1B**).

An *Arabidopsis* receptor complex was reported to control the maintenance of pollen tube integrity during its growth in the style, which is composed of pollen-specific CrRLK1L subfamily receptor-like kinases ANXUR1/2 (ANX1/2), Buddha's Paper Seal 1/2 (BUPS1/2) and LORELEI-like-GPI-anchored protein 2/3 (LLG2/3). This complex is localized in the apical membrane of the pollen tube and functions by perceiving the autocrine peptide ligands, rapid alkalinization factor 4/19 (RALF4/19) (Boisson-Dernier et al., 2009; Miyazaki et al., 2009; Ge et al., 2017; Mecchia et al., 2017; Feng et al., 2019; Ge et al., 2019b). The pollen tube of the *ralf4,19* double mutant also displays precocious rupture *in vitro* and inhibited growth in the transmitting tract, which is similar to that of the *llg2,3*, *bups1,2* and *anx1,2* double mutants. However, RALF4 induced the production of ROS in the pollen tube that stimulates the pollen tube growth and inhibits the pollen burst *in vitro* (Feng et al., 2019). An Arabidopsis *llg2,3* double mutant pollen tube exhibited reduced ROS levels and burst after germination *in vitro*, and the application of exogenous H_2_O_2_ rescued the rupture of the pollen tube (Feng et al., 2019). As suggested (Boisson-Dernier et al., 2013), the LLG-BUPS-ANX receptor complex functions upstream of RBOHH/J and regulates their activities to coordinate ROS production and Ca^2+^ homeostasis in regulating the pollen tube growth in the pistil. In this process, the activities of RBOHs are mediated by RopGEF-ROP downstream of ANX1/2 (Zhu et al., 2018; Feng et al., 2019) (**Figure 1B**).

The NADPH oxidases RBOHs that serve as the primary sources of apoplastic/cytoplasmic ROS in the pollen tube are widely studied in male–female interactions. However, little is known about the roles of ROS from other sources, such as the mitochondrion, peroxisome, and various oxidases. In addition hypoxia induces ROS production and *RBOHH* expression in plants (Pucciariello and Perata, 2017; Yamauchi et al., 2017). The transmitting tract provides a hypoxic surrounding for the pollen tube growth within it, which results from the restricted oxygen diffusion and active carbohydrate metabolism in the growing pollen tube (Goetz et al., 2017). We found evidence for this in the fact that the expression of ethanol degradation-related genes has changed in the pollinated stigma and style (Xu et al., 2013; Yue et al., 2014). Considering the availability of oxygen in the pollen tube, the ROS metabolism of the pollen tube growing in the transmitting tract might be different from that of the growing tubes *in vitro*.

## ROS Are Required for Pollen Tube Reception in the Female Gametophyte

The pollen tube grows into the micropyle and ruptures in a degenerated synergid to release two sperm cells that are ready for fertilization, which is under the control of the interaction between the pollen tube and synergid (Leydon et al., 2015). The pollination induces a ROS burst inside the embryo sac (Martin et al., 2013). It has been proven that the pollen tube rupture in the synergid is controlled by ROS from NADPH oxidases in the female gametophyte (Duan et al., 2014). FERONIA (FER), a universally expressed CrRLK1L family member, mediates the pollen tube rupture by inducing ROS generation at the entrance of the female gametophyte. Glycosylphosphatidylinositol-anchored protein LORELEI (LRE) and early nodulin-like protein functions (ENODLs) might be the co-receptors for FER signaling, which is also involved in ROS generation in the synergid (Duan et al., 2014; Li C. et al., 2015; Hou et al., 2016; Zhong and Qu, 2019). In the *lre* mutant, the ovule showed reduced levels of ROS, and the pollen tube revealed an overgrowth phenotype in the mutant ovule (Duan et al., 2014). However, the ectopic expression of LRE in the pollen tube could rescue the pollen tube rupture in the ovule of *lre* mutant (Liu et al., 2016). Thus, the LRE-FER signaling that was recovered could induce an instantaneous burst of ROS in the synergid cells that is adequate for pollen tube reception. The interactions between RAC/ROPs and FER and LRE indicate that RAC/ROPs mediate the activation of NADPH oxidase for ROS generation (Duan et al., 2010; Duan et al., 2014). Therefore, a FER-RAC/ROP-NADPH oxidase-ROS signaling pathway exists in the interactions between the pollen tube and female gametophyte (Li C. et al., 2015; Nissen et al., 2016). In addition, an ovule-expressed RALF peptide, RALF34, induces pollen tube rupture *in vitro.* RALF34 binds both BUPS1/2 and ANX1/2 *in vitro*, indicating that RALF34 may play its roles *via* the BUPS/ANX receptor complex (Ge et al., 2017). RBOHH- and RBOHJ-mediated ROS function downstream of the BUPS/ANX receptor complex that regulates pollen tube growth in the pistil (Boisson-Dernier et al., 2013). Thus, ROS may be involved in the RALF34-BUPS/ANX receptor complex signaling in the pollen tube rupture in the synergid cells (**Figure 1C**).

## ROS Trigger Downstream Responses

RBOH-derived ROS that mediate pollen tube integrity are required for either pollen tube growth in the pistil or pollen tube reception in the female gametophyte. Ca^2+^ and Ca^2+^-mediated protein phosphorylation functions in the activation of RBOHH and RBOHJ in pollen tube growth (Kaya et al., 2014). It has been suggested that Ca^2+^ binding triggers the production of ROS, which can also act on Ca^2+^ channels (Wudick and Feijó, 2014). However, the ROS-activated Ca^2+^ channels in the pollen tube are elusive. The cyclic nucleotide gated channel (CNGC) family functions as Ca^2+^ channels in pollen tube growth and guidance (Tunc-Ozdemir et al., 2013; Gao Q.-F. et al., 2016). The pollen tube of a *cngc7,8* double mutant shows a similar phenotype with that of the *rbohj,h* double mutant: bursting *in vitro* and sterility (Tunc-Ozdemir et al., 2013; Lassig et al., 2014). Thus, the activity of the CNGCs might be regulated by ROS in pollen tube growth, although experiments are required to test this hypothesis. The ROS-induced opening of the Ca^2+^ channels is required for pollen tube reception (Duan et al., 2014; Wudick and Feijó, 2014). The auto-inhibited Ca^2+^ ATPase 9 and CNGCs might be the downstream targets of ROS in pollen tube rupture (Schiøtt et al., 2004; Tunc-Ozdemir et al., 2013; Gao Q.-F. et al., 2016). ATUNIS1/2 (AUN1/2), a type one protein phosphatase, was identified to act downstream of ANX and RBOHH/J, and its activity is inhibited by ROS, which enables AUN1/2 to play its role as a negative regulator in pollen tube integrity (Franck et al., 2018). MARIS (MRI) is a plasma membrane-localized receptor-like cytoplasmic kinase that acts downstream of ROS and is mediated by both the ANXs and RBOHH/J in controlling pollen tube integrity and growth in the pistil (Boisson-Dernier et al., 2015; Liao et al., 2016). However, the manner in which AUN1/2 and MRI mediate the ROS signaling in pollen tube growth remains unknown (**Figures 1B, C**).

The regulation of pollen wall cytomechanics by ROS is suggested, e.g., ^•^OH is involved in the loosening of the pollen intine in the germination pore region that might facilitate the enlargement of the pollen volume during hydration (Smirnova et al., 2014). However, little is known about how ROS regulate the wall cytomechanics of the growing pollen tube. ROS upregulation of pectin synthesis, PME activity and pectin demethylesterification in the root and other tissues was reported (Messenger et al., 2009; Xiong et al., 2015). The pollen tube wall is enriched in pectins, and pectin methylesterase activity is critical for pollen tube integrity and its growth in the transmitting tract (Jiang et al., 2005). RALF4 not only induces ROS production in the pollen tube but alters the composition of the pollen tube wall, such as callose and pectin, which are correlated with the pollen tube integrity and growth (Mecchia et al., 2017; Feng et al., 2019). A *llg2,3* double mutant pollen tube showed reduced ROS levels and altered pectin and callose deposition at the tip wall of the pollen tube (Feng et al., 2019). Thus, ROS might be implicated in pollen tube integrity by regulating the metabolism of wall materials, such as pectin and callose (**Figure 1B**). Before the arrival of pollen tube at the synergid, the micropylar end of the synegid accumulates ROS that is controlled by FER (Duan et al., 2014; Li C. et al., 2015). The ROS in synergids might be involved in the development of a filiform apparatus, as suggested in phloem in which a ROS signal induces the formation of wall ingrowths in the transfer cells (Andriunas et al., 2013). After the arrival of the pollen tube, the high level of ROS at the micropylar end of the synegid might function in the regulation of pollen tube integrity by its implication in the metabolism of wall materials.

To facilitate the penetration of the pollen tube into the pistil tissues, cell wall modification and softening and cell separation in pistil tissues is required (Marsollier and Ingram, 2018). Hydroxyproline-rich glycoproteins (HRGPs), such as leucine-rich repeat extensins (LRXs), are localized at the pollen tube surface and in the intercellular matrix. It has been suggested that these proteins function to separate the cell walls of pistil tissues, by serving as lubricating functions for pollen tube growth in the pistil (Marsollier and Ingram, 2018; Sede et al., 2018). Stigma-specific protein 1 (STIG1), a cysteine-rich protein expressed in pistil tissues in tobacco and petunia, promotes pollen tube growth (Verhoeven et al., 2005; Huang et al., 2014). STIG1 controls the secretion of the HRGP-rich extracellular matrix and the ROS production of the pollen tube in both PI(3)P-dependent and LePRK2-dependent manners. There might be a linkage between the pistil factor-induced ROS elevation in the pollen tube and the HRGP secretion-facilitated pollen tube growth in the pistil tissues. *Arabidopsis* GRIM REAPER (GRI) is a secreted protein that is similar to STIG1. GRI promotes the superoxide production that triggers cell death (Wrzaczek et al., 2015). Thus, STIG1-promoted pollen tube growth in the pistil tissues might function *via* mediating ROS-induced PCD of the transmitting tract, as in the rice style (Xu et al., 2017). Rice OsCNGC13, a pistil-preferentially expressed CNGC member, plays roles in Ca^2+^ signal-inducing pistil PCD, which facilitates the penetration of the pollen tube in the pistil (Xu et al., 2017). Considering that pollination is an inducer for the PCD of transmitting tissue (Wang et al., 1996; Xu et al., 2017), it is tempting to study whether the apoplastic ROS of the pollen tube growing in transmitting tissue could diffuse into the pistil tissue to trigger the OsCNGC13 activity and pistil PCD in rice (**Figure 1B**).

Redox regulation for thiol/disulfide-containing proteins is involved in sexual plant reproduction (Traverso et al., 2013). ROS that act as signaling molecules function in the oxidation of a critical cysteine thiol group within redox-sensitive proteins (Reczek and Chandel, 2015; Sevilla et al., 2015). Cysteine-rich peptides (CRPs) expressed in either male or female reproductive tissues and cysteine-rich proteins as receptor complex subunits, such as LRE family members (LRE and LLG1-3) (Liu et al., 2016; Feng et al., 2019), are involved in male–female interactions, as was recently reviewed (Zhong and Qu, 2019). The modified eight-cysteine motif in the LRE is required for pollen tube reception (Liu et al., 2016). Recently, the N-terminus of the RALF23 peptides was identified to be involved in the binding to LLGs to assemble the LLG-FRE receptor complex to regulate immune signaling (Xiao et al., 2019), but the functions of the C-terminal region of RALF23 peptides with the conserved cysteine residues are unknown (Ge et al., 2019a; Ge et al., 2019b). Whether ROS functions in the redox modification and activity regulation of the cysteine-rich peptides and proteins in male–female interactions merits further investigation.

## Are ROS Involved in Pollen Tube Guidance Growth and Fertlization?

There are less data about the roles of ROS in pollen tube guidance growth and fertilization. CRPs secreted from the female gametophyte as a signal are required for pollen tube guidance (Takeuchi and Higashiyama, 2012; Li H.-J. et al., 2015; Meng et al., 2019; Zhong et al., 2019). *Arabidopsis* pollen-expressed GPI-AP COBRA-LIKE 10 (COBL10) and its modification play roles in the guidance of pollen tube growth (Li et al., 2013; Cheung et al., 2014; Dai et al., 2014). The *Arabidopsis* COBRA-LIKE protein family harbors at least 12 conserved cysteine residues, and several intramolecular disulfide bonds in COBL10 were predicted (**Supplemental Data 1**). Whether ROS is involved in the thiol-based redox modification of these CRP proteins and these modifications function in pollen tube guidance growth await further study. In addition, small cysteine-rich EGG CELL 1 proteins secreted from the egg cell in *Arabidopsis* are necessary for sperm cell activation in male–female gamete interactions for fertilization (Sprunck et al., 2012; Cyprys et al., 2019). As mentioned previously, the cysteine residues in CRPs are the potential targets of ROS signaling; thus, the implication of ROS in pollen tube guidance and fertilization is expected by mediating the redox modification of the CRPs. Higher ROS levels in the central cell of the female gametophyte before fertilization has been reported in *Arabidopsis* (Martin et al., 2013). Cytosolic ascorbate peroxidase (cAPX) is a central component in the metabolism of ROS. Abundant cAPXs in rice egg cells were identified, indicating that ROS scavenging is required for fertilization (Uchiumi et al., 2007). Thus, an open question is how the ROS signaling in the gametes (egg and central cell) is implicated in male–female gamete recognition in fertilization.

## Future Directions

In recent years, ROS that function as critical signal molecules have resulted in significant advances in various stages of pollination, including the interactions between the pollen and stigma, pollen tube and transmitting tract, pollen tube and female gametophyte. However, there are still many gaps in understanding the ROS action as a signaling molecule in male–female interactions. For example, it remains to be studied whether ROS are involved in pollen tube guidance growth and fertilization. In most of the previous studies, ROS burst in pollen tube and embryo sac is generated by plasma membrane-localized NADPH oxidases. In fact, other subcellular compartments generate ROS in plant cells, such as cytosol, chloroplasts, mitochondria, and peroxisomes (Mignolet-Spruyt et al., 2016). However, the roles of these ROS sources in the male–female interactions during pollination are less known now. ROS homeostasis is under the control of diverse antioxidant system, such as thioredoxin and glutathione (Zhang et al., 2018). The mutations of *Arabidopsis* NADPH-dependent thioredoxin reductase A and glutathione reductase 1 disturb the transmission of male gametophyte, although the pollen development is normal (Marty et al., 2009). These indicate that the ROS homeostasis in pollen tube governed by thioredoxin and glutathione is critical for pollen tube growth in pistil or fertilization. Thus, the regulation of ROS homeostasis in male–female interactions can be expected.

## Author Contributions

All authors listed have made a substantial, direct and intellectual contribution to the work, and approved it for publication.

## Funding

This work is supported by National Natural Science Foundation of China (31770349), Youth Program of National Natural Science Foundation of China (31800267), and Major Research Plan from the Ministry of Science and Technology of China (2013CB945100).

## Conflict of Interest

The authors declare that the research was conducted in the absence of any commercial or financial relationships that could be construed as a potential conflict of interest.
