# Supplementary material for: ROS in the Male–Female Interactions During Pollination: Function and Regulation
Source: Front Plant Sci. 2020 Feb 28;11:177. doi: 10.3389/fpls.2020.00177 (PMC7059789; doi:10.3389/fpls.2020.00177)

1 Predicated intramolecular disulfide bonds in COBL10

(performed on the website http://clavius.bc.edu/~clotelab/DiANNA/main.html)

Predicted bonds	
58 - 421	PEMAHCNGIFM - SWQVVCNITKP
193 - 636	NDGWECPAAKR - FNGEECALPKH
206 - 311	GSMHVCCKRNP - SQVMNCQRKPA
207 - 453	SMHVCCKRNPK - CNTCACGCNDI
291 - 434	KDPSECLYSKA - QASRCCVSFSA
336 - 505	GKLPFCCKNGT - PKKLPCPDNCG
337 - 390	KLPFCCKNGTL - NPQYKCGPPVR
433 - 451	AQASRCCVSFS - VPCNTCACGCN
448 - 509	NSAVPCNTCAC - PCPDNCGVSIN
455 - 463	TCACGCNDIDT - IDTDTCNANSN
**Predicted connectivity**	
1-10, 2-20, 3-6, 4-15, 5-12, 7-18, 8-9, 11-14, 13-19, 16-17	

COBL10 Protein sequence

```
  1 MRAIDVKTGM KIPWDVRYSL SLFIFLSSIL FLSNGQDYGM PGEDGGGGAE
 51 PPPEMAHCNG IFMSYNFGSR EREYPHVKNV TAQSWAFKST AMIVNAGREE
101 LKGWQMFIGF RHKELIVSAT GATPMDGDYP LDASNGTTFV GSPNMDLKTS
151 IETAGDFTQI SANIEITGTL FGVSKAVTPM PRTIKLTNDG WECPAAKRKG
201 GSMHVCCKRN PKIKNKIGLK TKFAPRRYGD LNIVYDVLQS FDSNYLAQVT
251 IDNDNPLGRL DRWNLTFEWM RGEFINTMRG AYTHKKDPSE CLYSKAGQYY
301 KDLDFSQVMN CQRKPAISDL PPEKKEDNMT GKLPFCCKNG TLLPPIMDPS
351 KSRSMFQLQV FKLPPDLNRT ALYPPQHWKI DGVLNPQYKC GPPVRVDPSQ
401 FPDPSGLLAV TYAISSWQVV CNITKPKAQA SRCCVSFSAF YNNSAVPCNT
451 CACGCNDIDT DTCNANSNPL LLPPDALLVP FDNRTLKAKA WAKQNHMPVP
501 KKLPCPDNCG VSINWHVSTD YKNGWTARLT VFNWRDFAFE DWFVAIDMGK
551 AGPGYENVYS FNGTRVPPSN RTVIFQGLPG MNYLVGQVNG TNPLRDPPVP
601 GKQQSVISFT KKNIKGLNIP EGDGFPTKLF FNGEECALPK HFPKKSSGHR
651 RGISVSMSFV FATIAAFALM MD
```

2 Alignments of COBRA-like proteins in Arabidopsis

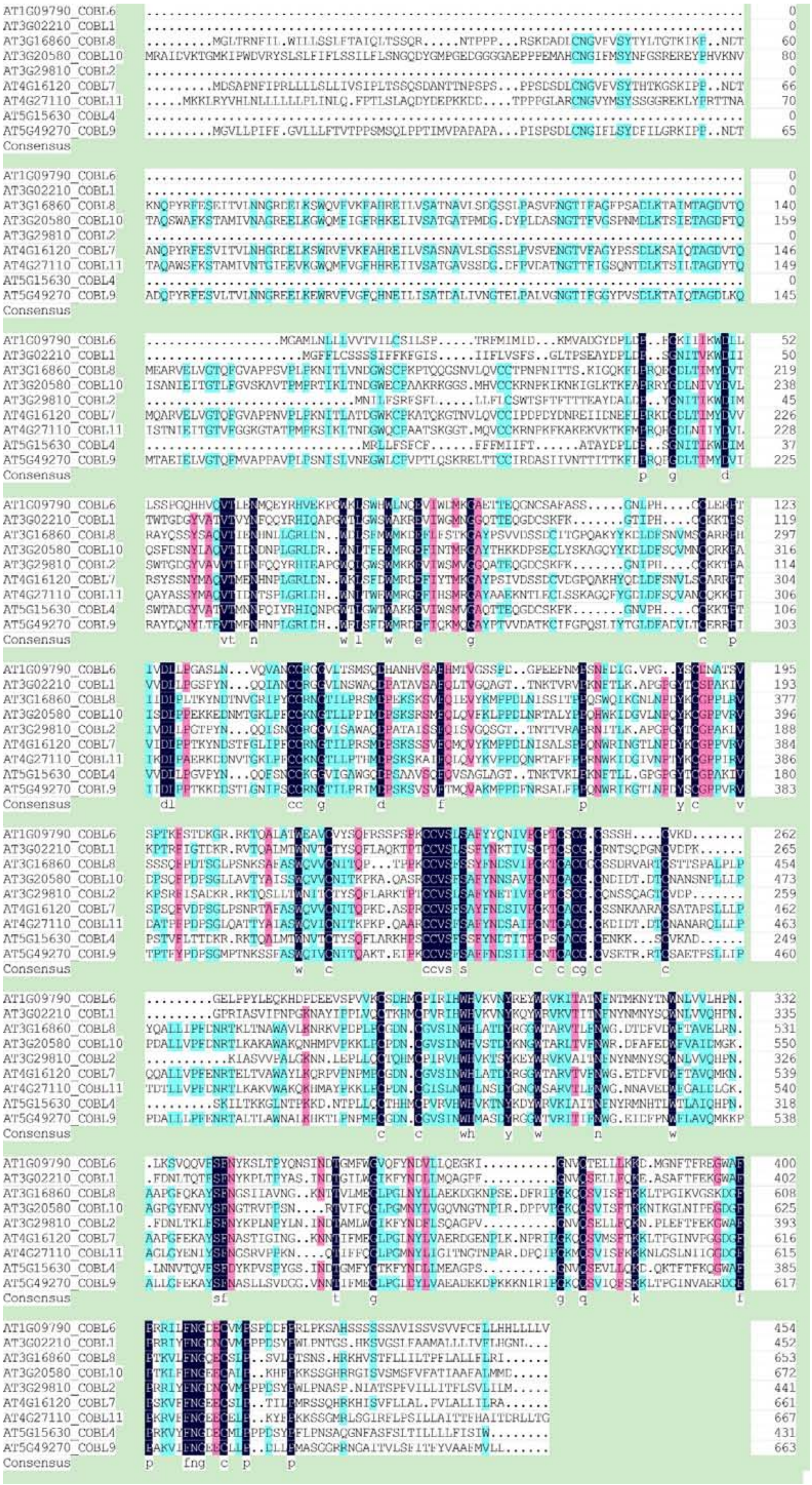

Supplement: Supplementary file 1 [file DataSheet_1.pdf]
